# Supplementary figures and images for: Faster emergence behavior from ketamine/xylazine anesthesia with atipamezole versus yohimbine
Source: PLoS One. 2018 Oct 29;13(10):e0199087. doi: 10.1371/journal.pone.0199087 (PMC6205597; doi:10.1371/journal.pone.0199087)

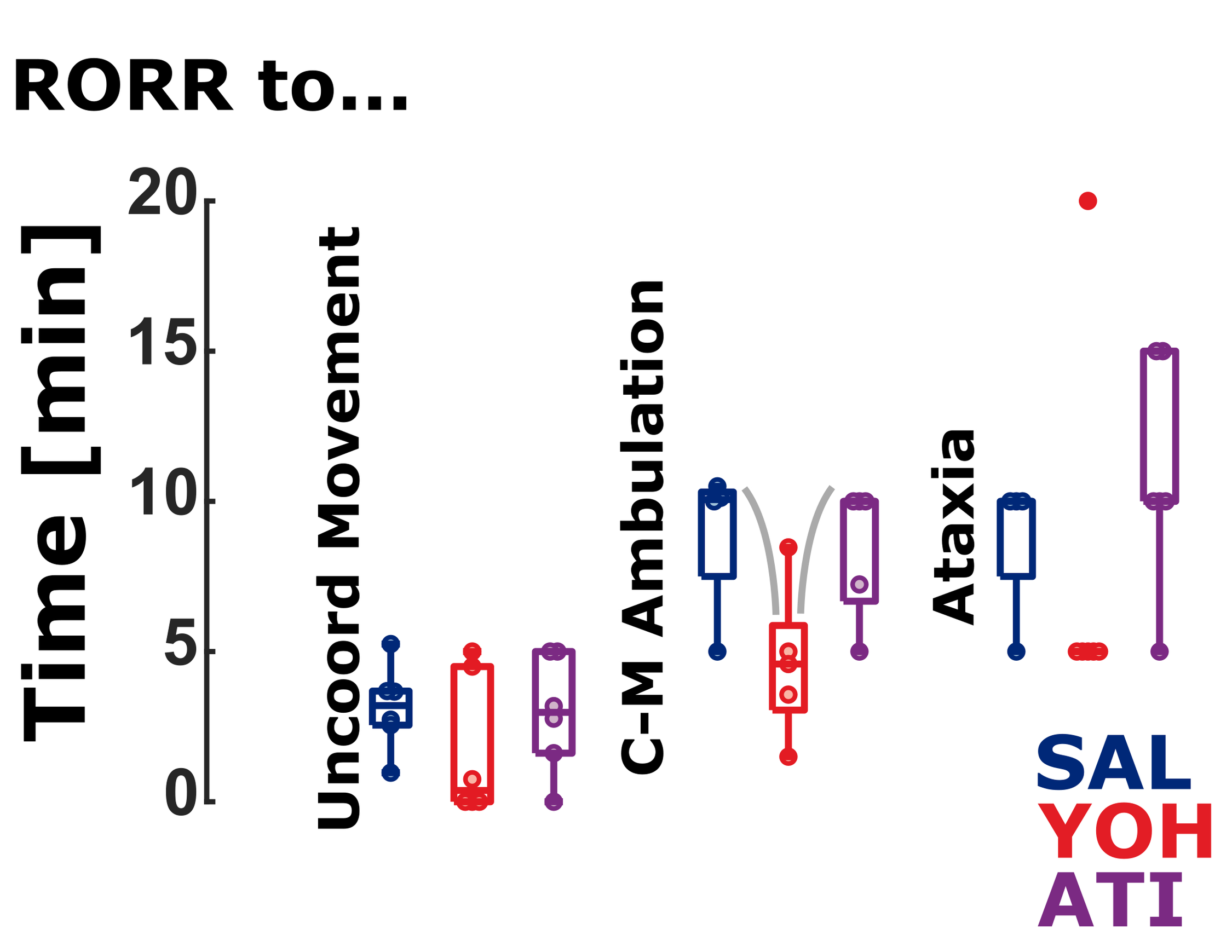

Supplement: S1 Fig — Latency from RORR to A) uncoordinated movement, B) cross-matched ambulation, and C) ataxia. There was no significant difference between these groups except for B) Mice from the YOH group had a significantly shorter time span from RORR to cross-matched ambulation. The solid lines between the boxes indicate significance. (TIF) [file pone.0199087.s001.tif]

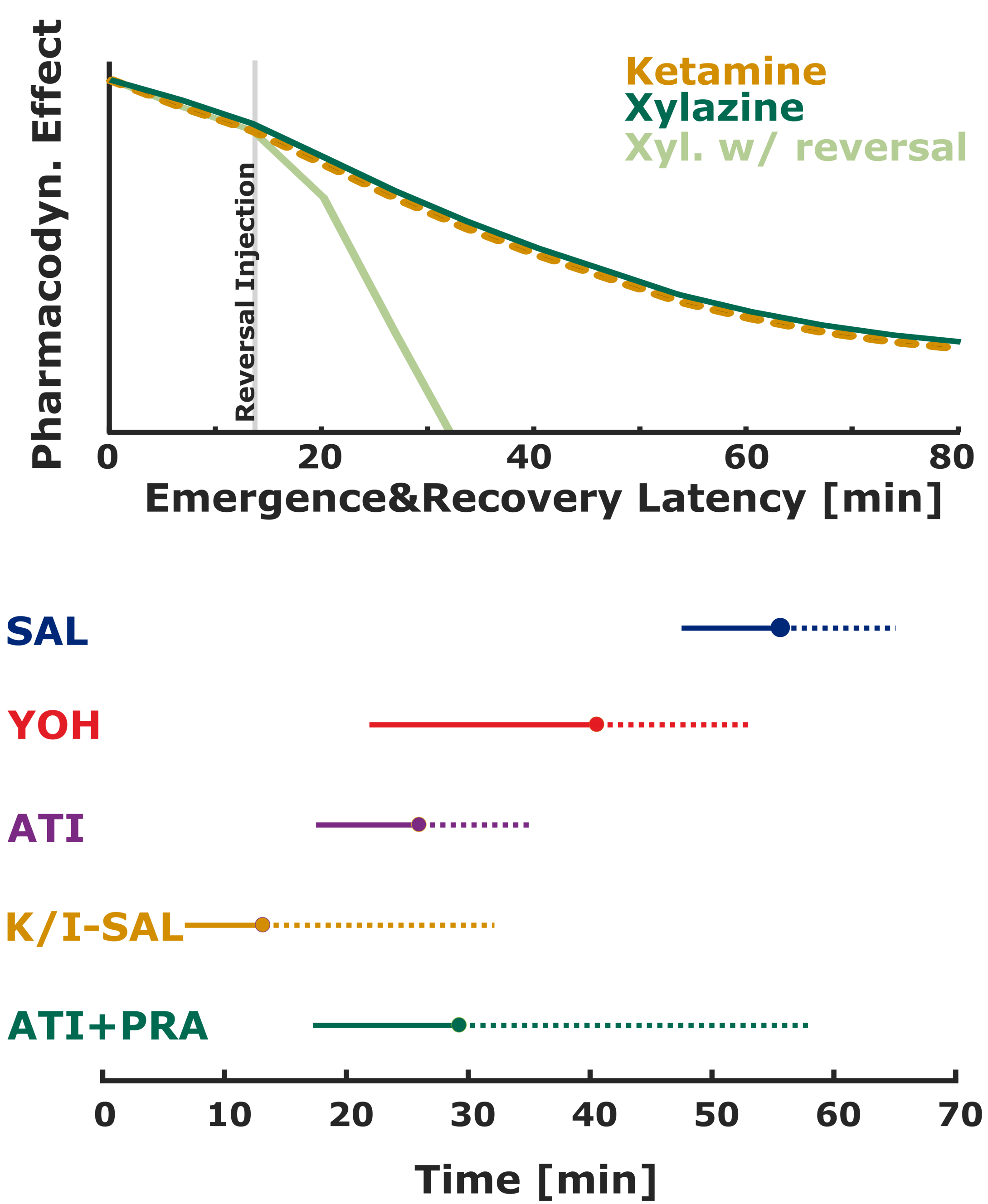

Supplement: S2 Fig — The upper graph is an idealized model that schematically depicts the expected pharmacodynamic effects (estimated overall effect of the drugs on the animal) of anesthetic agents over time (x-axis on the same scale as lower graph). Ketamine = yellow line, xylazine = green, xylazine with reversal agent = light green line. Mean latency for emergence period (whisker movement to RORR, solid lines) and recovery period (RORR to sticky dot notice, dashed lines) is plotted on the lower graph. SAL = blue, YOH = red, ATI = purple, K/I-SAL = yellow, ATI+PRA = green. Mean values are plotted. Vertical dashed gray line represents time of reversal agent injection. (TIF) [file pone.0199087.s002.tif]
